# An intersectionality-based policy analysis framework: critical reflections on a methodology for advancing equity

**DOI:** 10.1186/s12939-014-0119-x

**Published:** 2014-12-10

**Authors:** Olena Hankivsky, Daniel Grace, Gemma Hunting, Melissa Giesbrecht, Alycia Fridkin, Sarah Rudrum, Olivier Ferlatte, Natalie Clark

**Affiliations:** School of Public Policy, Simon Fraser University, Vancouver, British Columbia (BC) Canada; London School of Hygiene & Tropical Medicine, London, UK; Dalla Lana School of Public Health, University of Toronto, Toronto, Ontario Canada; Institute for Intersectionality Research & Policy, Vancouver, BC Canada; Geography, Simon Fraser University, Vancouver, BC Canada; Interdisciplinary Studies Graduate Program, University of British Columbia (UBC), Vancouver, BC Canada; Institute for Gender, Race, Sexuality and Social Justice, UBC, Vancouver, BC Canada; Faculty of Health Sciences, Simon Fraser University, Vancouver, BC Canada; School of Social Work, UBC, Vancouver, BC Canada; School of Public Policy, Simon Fraser University, Vancouver, BC Canada

**Keywords:** Intersectionality, Equity, Policy analysis, Reflexivity, Health

## Abstract

**Introduction:**

In the field of health, numerous frameworks have emerged that advance understandings of the differential impacts of health policies to produce inclusive and socially just health outcomes. In this paper, we present the development of an important contribution to these efforts – an Intersectionality-Based Policy Analysis (IBPA) Framework.

**Methods:**

Developed over the course of two years in consultation with key stakeholders and drawing on best and promising practices of other equity-informed approaches, this participatory and iterative IBPA Framework provides guidance and direction for researchers, civil society, public health professionals and policy actors seeking to address the challenges of health inequities across diverse populations. Importantly, we present the application of the IBPA Framework in seven priority health-related policy case studies.

**Results:**

The analysis of each case study is focused on explaining how IBPA: 1) provides an innovative structure for critical policy analysis; 2) captures the different dimensions of policy contexts including history, politics, everyday lived experiences, diverse knowledges and intersecting social locations; and 3) generates transformative insights, knowledge, policy solutions and actions that cannot be gleaned from other equity-focused policy frameworks.

**Conclusion:**

The aim of this paper is to inspire a range of policy actors to recognize the potential of IBPA to foreground the complex contexts of health and social problems, and ultimately to transform how policy analysis is undertaken.

## Introduction

In the field of health, numerous frameworks (e.g., sex and gender based analysis, health equity impact assessments) have emerged over the last fifteen years, all attempting to advance better understandings of the differential impacts of health policies and to produce inclusive and socially just health outcomes [1-6]. Despite progress made to date, there is still much work to be done to better understand how policy affects diverse populations, including precisely identifying who is benefiting and who is excluded from health policy goals, priorities and related resource allocation. As part of the ongoing efforts to move forward work in this field, there is a growing interest in the theory of intersectionality and its potential to improve current equity-driven health policy analyses [7-10]. To date, however, this potential has not been realized, largely due to the fact that few methods have been developed to operationalize intersectionality in the context of health policy.

In this paper, we describe an innovation for policy analysis that fills this gap: the Intersectionality-Based Policy Analysis (IBPA) Framework. Developed and refined through an iterative, participatory process inclusive of multiple sectors, IBPA is intended to capture and respond to the multi-level interacting social locations, forces, factors and power structures that shape and influence human life and health. Its aim as a policy tool is to better illuminate how policy constructs individuals’ and groups’ relative power and privileges vis-à-vis their socio-economic-political status, health and well-being. Significantly, we also present a synthesis of seven health-related policy case studies based on this Framework. The purpose of this synthesis is not to provide a detailed overview of each case study, which is available elsewhere [11] but rather to clearly and succinctly distill the value and benefit of conducting IBPA in relation to these diverse areas of policy. As such, the analysis of each case study is focused on explaining how IBPA: 1) provides an innovative structure for critical policy analysis; 2) captures the different dimensions of policy contexts including history, politics, everyday lived experiences, diverse knowledges and intersecting social locations; and 3) generates transformative insights, knowledge, policy solutions and actions that cannot be gleaned from other equity-focused policy frameworks. The aim of this paper is to inspire policy practitioners and actors to recognize the potential of IBPA to foreground the complex contexts of health and social problems, and ultimately to transform how policy analysis is undertaken.

### Intersectionality

Rooted in a long and deep history of Black feminist writing, Indigenous feminism, third world feminism, and queer and postcolonial theory [12-16], intersectionality has emerged as a widely respected, albeit variously defined research and policy paradigm [17]. Nevertheless, there are a number of central tenets that capture the unique nature of this paradigm. These are:

- human lives cannot be reduced to single characteristics;
- human experiences cannot be accurately understood by prioritizing any one single factor or constellation of factors;
- social categories/locations, such as ‘race’/ethnicity, gender, class, sexuality and ability, are socially constructed, and dynamic
- social locations are inseparable and shaped by interacting and mutually constituting social processes and structures, which, in turn, are shaped by power and influenced by both time and place; and
- the promotion of social justice and equity are paramount [8,11].

Intersectionality encourages critical reflection that allows researchers and decision makers to move beyond the singular categories that are typically favoured in equity-driven analyses (e.g., sex and gender in sex and gender based analysis) and also beyond the kind of enumerated list of determinants of health often found in health impact assessments to consider the complex relationships and interactions between social locations such as Indigeneity, sexuality, gender expression, immigration status, age, ability and religion^a^. This enables an examination of the simultaneous impact of and resistance to systems and structures of oppression and domination, such as racism, classism, sexism, ableism and heterosexism [8]. Intersectionality is concerned with bringing about a conceptual shift in how researchers, civil society, public health professionals and policy actors understand social categories, their relationships and interactions. It requires a consideration of the complex relationship between mutually constituting factors of social location and structural disadvantage so as to more accurately map and conceptualize determinants of equity and inequity in and beyond health [18].

An ongoing challenge in advancing this body of work is the further development of explicit and user-friendly methods that can more effectively translate intersectionality theory into practical approaches to be understood and used by decision makers and policy researchers. Taking on an intersectionality study/analysis can be incredibly intimidating. Bowleg [19] states, although intersectionality theory provides a conceptually solid framework with which to examine the social locations of individuals and groups within the broader interlocking structures of power relations [20,21], the methodological choices available to do so and/or guidance offered on *how* to do so are severely limited [22-26]. In response to this gap, a handful of tools have recently been developed for applying intersectionality to public policy [8,25,27-29] which have started to illuminate the potential of intersectionality. None to date, however, have specifically been developed for health and health-related policies and programs, making the IBPA detailed below, a significant contribution to the literature.

## Methods

The Intersectionality-Based Policy Analysis (IBPA) Framework and corresponding case studies were developed in an iterative, participatory process. Beyond the input of the authors, the final Framework reflects the feedback received from emerging and established scholars in the field within academic, governmental and community settings. In particular, it responds to feedback from policy actors across provincial and federal departments who increasingly report having ‘lens fatigue’ navigating an increasingly numerous terrain of policy lenses focused on various factors and considerations such as gender, geographic location, illness status, age, and ability.

Based on a series of meetings and peer feedback, as well as on critical reflection into current gaps and trends in equity-promoting public policy analysis, a draft IBPA Framework was collaboratively developed to guide the development of the case studies. This draft was further revised near the completion of the case studies, as the intention of the group was to engage in an ongoing process of refinement to ensure that the IBPA is a usable and practical guide for policy analysis.

**Figure 1:**
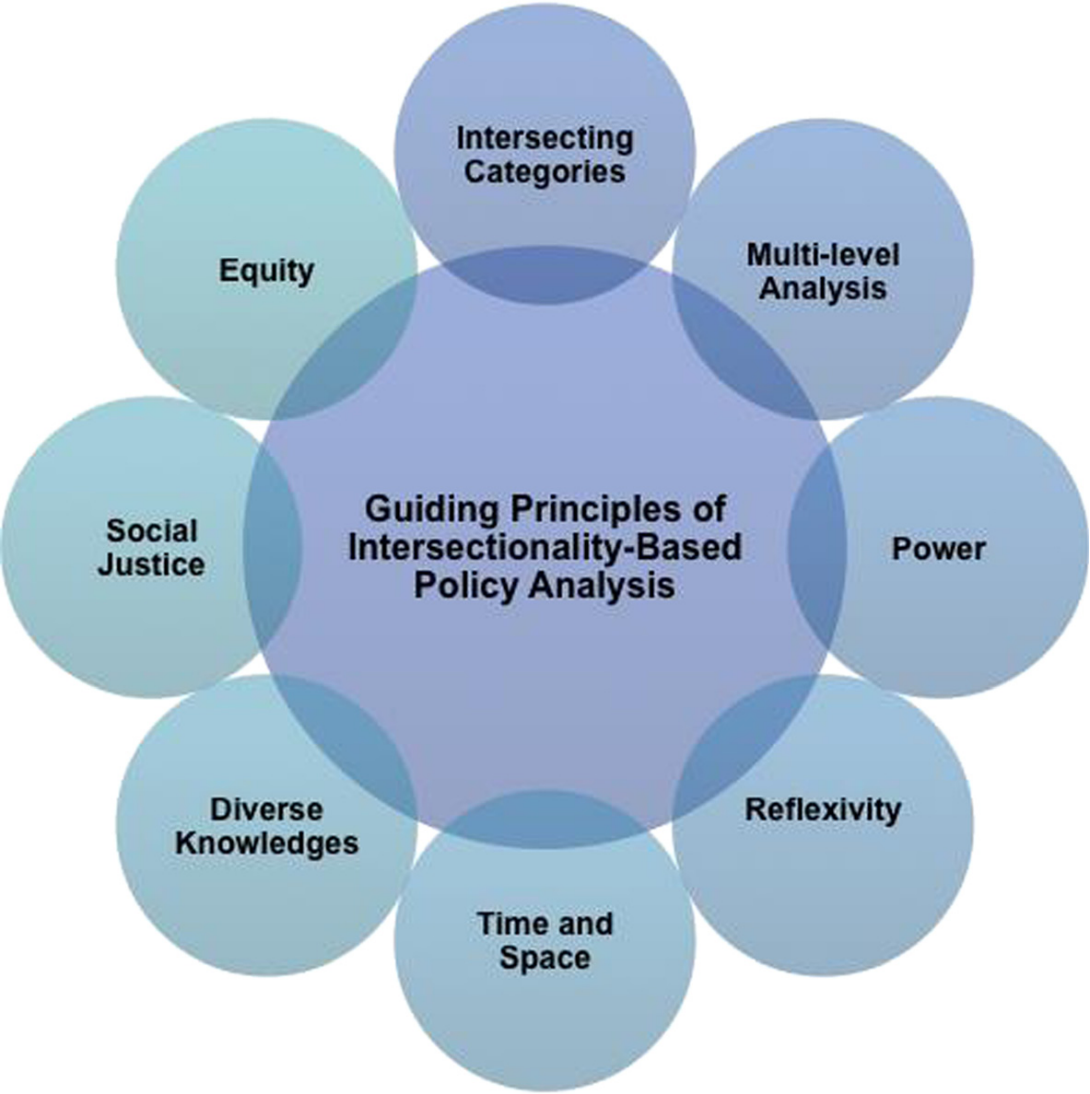
**Guiding principles of Intersectionality-Based Policy Analysis.**

**Figure 2:**
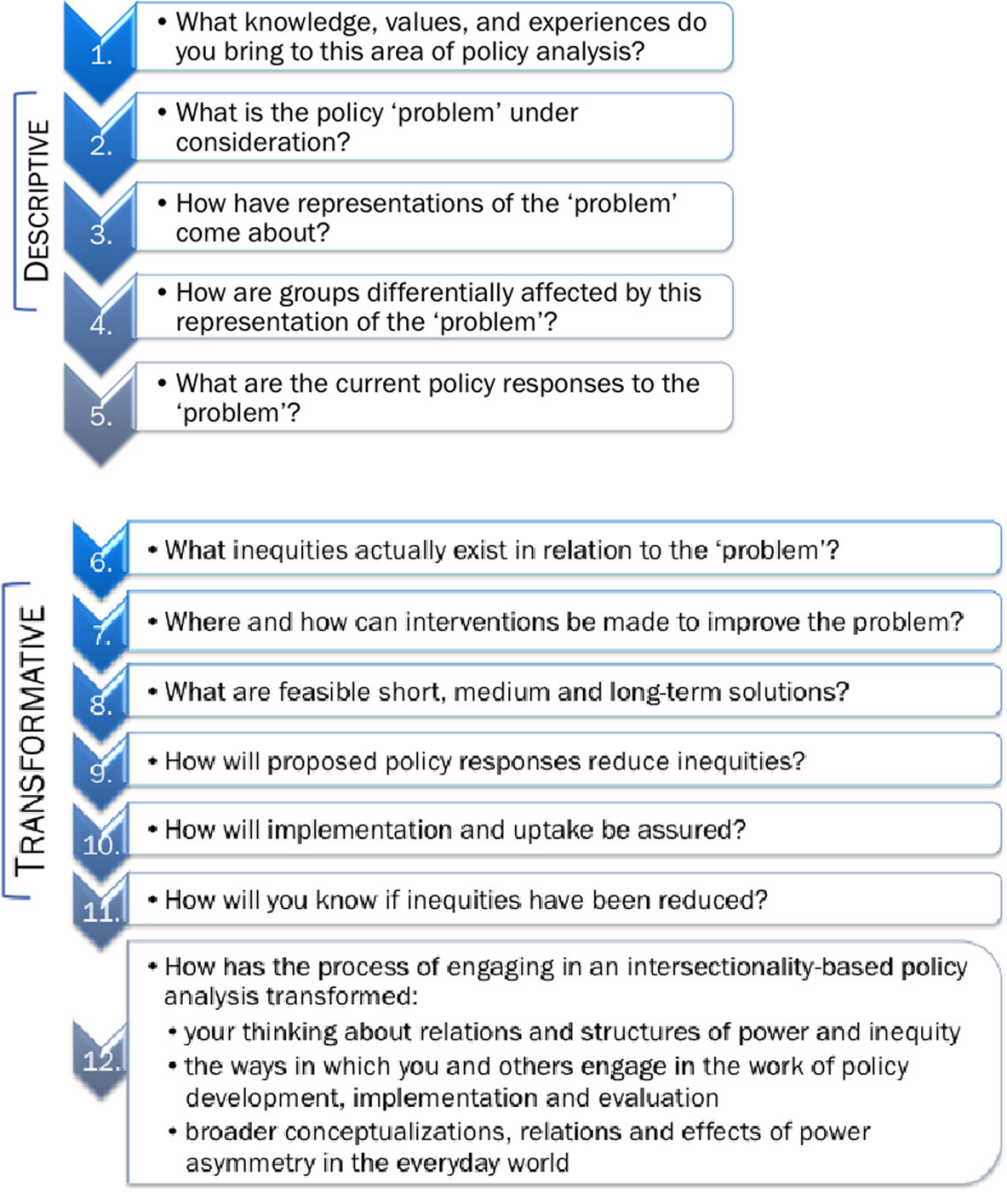
**Descriptive & transformative overarching questions of IBPA.**

The IBPA Framework has two core components: a set of guiding principles (see Figure 1) and a list of 12 overarching questions to help shape the analysis (see Figure 2). The guiding principles are intended to ground the 12 key questions, including their supporting sub-questions, in order to ensure that each is asked and answered in a way that is consistent with an intersectionality-informed analysis. ^b^Put succinctly, the principles are designed to be used in concert with the questions.

The questions are divided into two categories: *descriptive* and *transformative*. Their combined effect is intended to expand and transform the ways in which policy problems and processes are understood and critically analyzed in order to ensure fine-tuned and equitable policy recommendations and responses. The first set of *descriptive* questions is intended to generate critical background information about policy problems in their full context, with specific attention to the processes and mechanisms by which policy problems are identified, constructed and addressed. Their purpose is to reveal assumptions that underpin existing government priorities, the populations targeted for policy interventions, and what inequities and privileges are created by current policy responses. The second set of *transformative* questions is intended to assist with the identification of alternative policy responses and solutions specifically aimed at social and structural change that reduce inequities and promote social justice. The questions in this section prompt users to consider actions that will ensure meaningful uptake of equity-focused policy solutions as well as the measurement of the impacts and outcomes of proposed policy responses.

Simplicity and flexibility are key features of the Framework. While some users may ultimately ask all 12 questions to help guide their analysis, others may focus on certain questions, tailoring them to specific policy contexts. Some questions may be more or less relevant depending on the policy under examination, its history, and its stage of development and implementation. At the same time, it is critical that the questions be grounded in key intersectionality principles to ensure IBPA’s transformative effects on how policy problems and issues are understood and responded to.

Each of the case studies, briefly described in the following section, utilizes IBPA to analyze key health and health related policy areas. Collectively they demonstrate the added value of engaging with intersectionality for analyzing social and health inequities. At the same time, each author applies the IBPA in very different ways, demonstrating the flexibility of this Framework. However, they each also make explicit - concretely and persuasively – why IBPA allowed them to discover new insights and knowledge about particular policy problems.

## Results and discussion

To date, the authors of the IBPA Framework have applied this mode of critical policy analysis to seven different health policy fields. Elsewhere [11] these policy examples are presented in full detail. In this paper, however, we highlight what we consider the most salient components of the IBPA and use these to frame the discussion of each unique case study. Our goal is to clearly and succinctly demonstrate – across a diversity of health and health-related issues - the advancements that can be realized by using intersectionality in the analysis of policy.

The first component that each policy example discusses is the *structural innovation* of the IBPA Framework. This component is characterized by three defining elements of an IBPA-informed analysis: the interrogation, using diverse sources of information and knowledges, of the implicit assumptions underpinning policies; the attention to historic developments and contemporary framings of social issues and policy problems; and the self-reflexive method for capturing complex multi-dimensional power dynamics that shape everyday lived experiences.

The second component that the case studies highlight is the *transformative effects* of IBPA**.** This part of the discussion seeks to demonstrate how an IBPA generates new perspectives and insights about policy issues and affected populations. As all the authors show, new knowledge and evidence has significant potential to disrupt and challenge the status quo, including the most progressive approaches to policy development, implementation and evaluation. Finally, the case examples also illuminate why an IBPA provides directions for renewed advocacy efforts aimed at social change and social justice.

The first two case studies focus on policy issues typically understood as highly gendered phenomena. Both authors, however, draw on IBPA to illustrate the importance of multiple social locations and structures of power, including but not limited to gender, that influence the availability and delivery of health services. To begin, Rudrum examines current maternity care policy, revealing inequities in access to high-quality appropriate care for differently situated women across geography, ethnicity, Aboriginal identity, and socioeconomic status. In the process, this author challenges the idea that there are fixed norms or standards in the care that women require in pregnancy and childbirth. Next, Giesbrecht focuses on palliative care policy, revealing the current inequities in access to services and supports, and demonstrating the extent to which ‘choices’ at the end of life by those who need and provide care are inextricably linked to interactions between socioeconomic status, service provision, cultural discourses, and emotional, spiritual and relational factors infused with physical and social aspects of place.

Three of the case studies specifically focus on issues relevant to Aboriginal health. Hunting’s examination of Fetal Alcohol Spectrum Disorder (FASD) shows why Aboriginal populations continue to experience health inequities in relation to current policies. She argues that a sole focus on women as a category, a narrow conception of risk, and a lack of attention to intersecting processes of oppression within FASD policy discourse undermine the development of IBPA-informed policy processes and reforms that can more effectively address the experiences, needs and perspectives of diverse populations affected by substance use. Second, in reviewing policy processes of the Kelowna Accord – an Aboriginal health policy initiative in Canada that was developed but never implemented – Fridkin demonstrates how IBPA can be applied to issues in Aboriginal health policy to promote the inclusion of Aboriginal peoples and knowledges in policymaking processes, which may contribute to agendas of decolonization. Fridkin illustrates how IBPA can be used to analyze not just policies themselves, but policy processes, thus highlighting the potential of IBPA to expand what is typically constituted as policy analysis. Third, using an IBPA lens, Clark shows that even policies that forefront Aboriginal needs fall short because they often fail to consider the multiple and intersecting layers of Indigenous identity, such as age, rurality, gender-expression and experiences of trauma, including interactions with multiple policy systems. Clark’s contribution is also important in that she draws significant parallels between intersectionality and Indigenous ways of knowing, while raising critical questions about the relationship between IBPA and Indigenous epistemology.

The final two case studies in the collection tackle various issues relating to HIV. First, Grace draws on IBPA to advance understandings of complex issues facing sexual minority populations by considering both current understandings and testing technologies surrounding HIV and the criminalization of HIV non-disclosure. He makes a persuasive argument for using IBPA to advance an equity-focused understanding of the ‘problem’ of HIV transmission that places front and centre the structural drivers that produce differential vulnerabilities among affected populations. Lastly, Ferlatte uses an intersectionality lens to evaluate HIV prevention funding for gay men. The examination includes consideration of discourses around HIV, funding application processes and funding decision outcomes. His analysis highlights the structural barriers involved in securing support for HIV prevention. Importantly, Ferlatte discusses possible alliances with other groups to work for policy change rooted in understandings of the power dynamics that currently shape the HIV funding system.

### Case 1: Maternity care

In October 2012, a labouring woman in the Ottawa-Carleton Correctional Institute in Ontario Canada was denied care and moved to segregation, where she gave birth to a breech baby unattended, after hours of labour. She had been checked by prison nurses who believed she was in ‘false labour.’ A minister of parliament called on to respond to the case described it as similar to an unplanned home birth, clearly overlooking the power disparities that contributed to the failure to provide care (CBC). Canadian policy makers and care providers agree that pregnant women should have choice, autonomy, and control over their health care, but, as this example demonstrates, experiences of care are in fact characterized by inequities related to social position and geographic location. While this scenario may seem exceptional, both national and provincial policy documents acknowledge a crisis in maternity health care [30]. This case study reviews the 2004 report, “Supporting Local Collaborative Models for Sustainable Maternity Care in British Columbia” by BC’s Maternity Care Enhancement Project [31] and two documents published as a result of this report, “Aboriginal Maternal Health in Canada: A Toolbox” (BC Aboriginal Maternal Health Project) [32], and the “Obstetric Guideline 19: Maternity Care Pathway” (BC Perinatal Health Program) [33].

### Structural innovation

Explicit attention to history and context is inherent in the IBPA principles on *Time and Space* and *Diverse Knowledges*. Applying these principles to the report yielded two major critiques: first, that human resource shortages are addressed in a manner that reinforces physician privilege while failing to contest gendered and racialized power imbalances within the health care professions; and second, that the approach to difference among maternity care clients does not adequately address differences among women or health inequities.

The first critique was generated through an examination of the history of midwifery and how the marginalization of midwives and their care negatively affects maternity care clients. Midwives have had to advocate for their profession to be formally recognized and publicly remunerated, and their presence in BC and elsewhere, has not always been welcomed by obstetricians or by other doctors providing maternity care, even though they attend births at home as well as in hospital, and see their clients more frequently and for longer visits than is typical for physicians. Addressing one group of providers’ concerns (e.g., physicians) shapes access to quality care, by promoting growth in provider group while restricting growth in another in a way that does not coincide with the needs of birthing women. Since choice in provider type and birth location is considered an important element of quality care, and since midwifery care is so unevenly available outside of urban areas, failing to address midwifery’s low numbers is also a failure to address a gap in quality service provision.

Second, the IBPA Framework helps orient policy to the concerns of people in their everyday lived experiences. IBPA encourages a focus on how groups are represented and conceptualized, through questions such as *What differences, variations and similarities are considered to exist between and among relevant groups?* An IBPA revealed that in the case of BC’s maternity care recommendations, the talk about diversity sounded hollow specifically because inequities that currently exist in maternity care provision and maternal health outcomes were not adequately considered. For example, challenges for rural women seeking care were alluded to but not adequately addressed. In comparison, an IBPA brings to the fore the lack of access to comprehensive and appropriate maternity care in rural and small communities. It also highlights the intersections with ethnicity: Aboriginal communities, including reserves, are often rural, and smaller communities have less access to health care decision-making bodies [34]. Refugee women also often have social and health concerns that can make pregnancy a uniquely vulnerable time [35]. Age also is an important intersection as young single women are often subject to social stigma, and are susceptible to risk labeling and accompanying surveillance and interventions.

Within the report and guidelines, while it is noted that health problems in pregnancy are related to addiction, experience of intimate partner violence, youth and poverty, these different factors are mostly presented as if affected women are part of a cohesive group. At the same time, the concerns of these women are also individualized as ‘lifestyle’ issues. This process of creating risk groups or individualizing social problems is relevant to another sub-question of IBPA question 4, *How do the current representations shape understandings of different groups of people?* Despite the good intentions of including guidelines related to various social factors, the potential benefit of these recommendations to groups experiencing health inequities is diminished by this tendency towards creating risk groups and individualizing health concerns whose dimensions are largely social. IBPA attends to the patterns and differences among affected women by locating them in context of systems of power, and this focus on differentials would travel throughout the policy process on maternity care.

### Transformative potential

Despite identifying ‘women-centred care’ as an important model for maternity care, the report does not elaborate on recommendations related to health inequities or on the range of needs of women in British Columbia. A women-centred approach is valuable in identifying that women should have a degree of choice, autonomy and control regarding their care and birthing practices. However, from an IBPA perspective, the model presented did not address how choice and autonomy are constrained by power systems of privilege and oppression.

Reviewing policy using the IBPA tool, with its ability to better address issues of power and inequity, a number of benefits for maternity care policy and delivery in BC can be realized. At the level of tools for care providers, such providers working with the broader population would benefit from information about issues including lack of local care, teen pregnancy, and addiction, for example, presented in a way that is not stigmatizing would benefit providers working with the broader population.

While policy in this area tends to treat women as a generic group, in practice, women are a diverse group who vary in their approaches to pregnancy, their health care needs, and their life circumstances; to ensure equitable access to quality care, maternity care policy needs to attend to the differences among women. This would include moving away from stigmatized understandings of ‘groups requiring additional care’ or vulnerable women, by starting from an understanding that there is not one fixed norm for the care women may require in pregnancy.

### Case 2: Palliative care

Reflecting a demographic trend witnessed in many nations, Canada is experiencing rapid population aging. This increase raises many concerns for health care planners and administrators, particularly in regard to the impending increased need for palliative care. Within Canada, this is offered across a range of sites, including nursing homes, acute care hospitals, respite facilities, and hospices by a variety of providers who can include family doctors, nurses, specialists, community volunteers, spiritual leaders, and family members [36]. However, reflecting neoliberal and social trends experienced in much of the global north, the ‘place’ where palliative care occurs in Canada is increasingly moving away from hospital settings and into the community, especially the home [37,38].

Currently, over 259,000 Canadians die each year; however, only 15 percent access palliative care services prior to death [39]. This statistic raises many concerns regarding the awareness, accessibility, and meaningfulness of palliative services for dying Canadians and their families [39]. Given the rapidly aging population and that a large percentage of dying Canadians, including British Columbians, and their caregivers are not accessing adequate palliative care, it is clear that a timely and significant need exists to enhance existing palliative care services and supports.

### Structural innovations

The diversity of participant experiences explored in this analysis was exceptionally vast as *everyone*, at some point in some way, will experience death and dying. Considering this, the potential diversity that exists among this population group may seem daunting for researchers who wish to employ intersectionality-based analyses. However, the structured guidance offered by the IBPA Framework was effective by uniquely guiding the researcher via particular questions and prompts, while simultaneously permitting flexibility *and* embracing complexity. For example, the *descriptive* questions prompt the reader to identify the context and what the policy ‘problem’ is. The ‘problem’ explored in this case study involved examining current BC palliative care policy that is directed towards supporting more British Columbians to die in the home, rather than in formal institutions, such as hospitals. However, it was the selected descriptive question that asks *How are groups differentially affected by this representation of the ‘problem’?* that provided the spring board for this case study analysis.

Much caregiving research tends to focus on the gendered nature associated with this role, however, because the Framework emphasizes that analyses must be anchored in the everyday lives of those the who the policy and resulting programs aim to serve, it embraced the diversity that actually exists among those in need of palliative care services. For example, as caregiving is generally seen as a ‘woman’s’ issue, the IBPA Framework revealed that gender is not necessarily the most important variable when considering needs and access to palliative care supports. More specifically, it may be one’s geographic location of residence, housing status, or access to social networks that *together* create a greater impact in shaping experiences of palliative caregiving, than simply being a woman. Additionally, findings revealed that recipients of palliative care are not a homogenous population group either, but rather carry a range of needs in regard to the types of palliative care supports they require. Furthermore, commonalities across groups also become visible due to the multi-dimensional lens of the Framework. For instance, the Haida people’s spiritual preference to not have a death occur in the home, those with insecure housing status, or those who are dying and do not have access to a family caregiver would *all* benefit from directing palliative care efforts towards enhancing meaningful access to palliative care supports *outside* of the home, for example by creating more hospice houses. Overall, the IBPA Framework provided a map for employing an intersectional approach to palliative care policy by providing valuable suggestions regarding where to begin (i.e., descriptive questions) and ultimately, where to go (i.e., *transformative* questions) during the analytic process.

### Transformative effects

In this case study, the IBPA Framework enhanced the visibility of those who are generally not acknowledged within the palliative care policy realm. Its application revealed that some groups face higher barriers in accessing supports and experience greater stresses and burdens in regard to having to provide informal palliative care in the home than others. For example, those who are located in rural and remote areas in BC, who are at great distances from services, who are socially isolated or stigmatized, and who may be complexly located under any of the existing arms of oppression (e.g., cultural minorities and/or First Nations, among other groups) face greater barriers to accessing palliative supports, and for the care recipient, achieving a death with dignity. On the other hand, this analysis also exposed characteristics of those who are situated in relatively privileged social and physical positions, for whom such policies are working - namely, those who have a relatively predictable prognosis and middle to high class status, who are located near a larger urban/town area, are home owners, and socially connected, married, and/or have an educated (preferably with a medical background) woman friend or family member who is healthy, willing, capable and available to take time to provide care in the home. Thus, using the Framework disrupted the common policy discourse that tends to assume that those in need of palliative care are a homogenous group of middle class, Anglo-European (white western), British Columbians who have safe and secure housing and live in nuclear family structures.

Generally, BC’s ‘one-size-fits-all’ approach to palliative care is tailored to a ‘standard person’, who arguably does not exist. Although current palliative care policy is directed towards assisting palliative care to take place in the home, the site of the home for palliative care may, or may not be, a viable and desirable option. The Framework uncovered the complexity of this issue and revealed that the preference for the home as a site for palliative care was intertwined with access to outside formal supports, spiritual beliefs, housing security and associated costs. More specifically, the findings point to the home as a highly contested site for palliative care, one characterized by intersecting political, cultural, economic, social, geographic and historical dimensions. By unpacking the policy directive towards enhancing supports for palliative care in the home, it also becomes apparent that the house, home and family have become conflated in the policy realm and are based on an ideologically laden perspective where families are seen as white, middleclass, heterosexual and nuclear.

Two principles of the IBPA Framework are *Social Justice* and *Equity*, and in order to address these principles, avenues for advocacy must be acknowledged. Explicitly from this case study, findings reveal valuable information that can be used to inform policy decision makers on directions and ways to provide more meaningful, equitable, and inclusive palliative care supports and services. More implicitly however, this case study casts a spotlight on a branch of health care that too often is undervalued and overlooked. This may simply be due to our society’s contemporary western view of death and dying, which has been characterized by some as being in ‘death denial’ [40-42]. Western health care delivery is characterized as being both highly curative and bio-medical in nature and, thereby, more interested in healing the bio-physical body than in addressing the psycho-social, cultural, and spiritual needs of the dying and their family members [42-44]. Advocacy is needed to advance palliative care policy in BC. Here, the valuable work of community hospice organizations, together with citizen advocacy, has the potential to assist with minimizing the cultural and social taboos around death and dying prevalent in both our society’s psyche and the Canadian health care system [42].

### Case 3: Fetal Alcohol Spectrum Disorder

Critical analysis of policy addressing Fetal Alcohol Spectrum Disorder (FASD) in Canada is particularly pressing given increasing health and social inequities, increased evidence of substance use among certain populations and increased public attention to FASD as a “a national public health, education, economic, and social concern” [45]. Recent critical analyses have highlighted the failure of FASD policy in Canada to account for the historical, structural and social contexts that situate substance use. Consequently, substance ‘users’ have been framed as the ‘problem’ requiring government intervention [46,47]. Converging with such constructions is the prevailing assumption, permeating the media, FASD prevention campaigns and public discourse, that FASD is predominantly an ‘Aboriginal problem’ [48-50]. Importantly, an IBPA Framework provides an innovative structure to examine how such discourse can reinforce relations of equity for people who use substances, while also providing transformative opportunities to rectify such tendencies.

### Structural innovations

This case study reveals how FASD-related policy (and research) to date have consistently perpetuated certain assumptions of who is affected and how (e.g., that FASD is a problem of Aboriginal mothers). The analytical guidance provided through the overarching questions of the IBPA Framework problematized such assumptions of what the problem is and who is affected. For example, asking *how* representations of the ‘problem’ of FASD have come about reveals the research and policy discourse surrounding FASD as often reflecting gaps, biases, and discriminatory assumptions. Pursuing this question can reveal, for instance, that: a) FASD-related research has historically focused on particular Aboriginal reserve communities where substance use rates were known to be elevated, to the exclusion of research that could reflect the prevalence of FASD within and across Aboriginal and non-Aboriginal populations; and b) the diagnostic indicators of FASD, and the identification of mothers who use substances have been argued to be racialized. Acknowledging this entrenchment of discriminatory practices can allow for policy actors to resist and reframe what the ‘problem’ is.

The IBPA Framework also allows one to ground their analysis with the question that asks*: What knowledge, values and assumptions do you bring to the area of policy analysis*? This acknowledges that all stages of policy processes and policy analyses occur are situated within intersecting social locations and contexts experienced by the analyst. Being reflexive as to ones assumptions about particular policy problems and what types of evidence and knowledge one considers valid allows for possible gaps and limitations in policy response to be revealed. This is particularly relevant to FASD-related policy, which has often reinforced dominant constructions of FASD as an issue of ‘Aboriginality’ while inadequately addressing the contexts of substance use. The critical reflection encouraged by IBPA in this case study is a necessary starting place in reforming discriminatory assumptions and practices, while better understanding and addressing the conditions situating FASD.

Importantly, IBPA guidance allows for the intersectional contexts of both maternal substance use and diagnosis of FASD to surface. The guiding principles that ground the questions are central to this. For instance, the principle of *Intersectional Categories* recognizes that looking at policy populations via singular categories is inadequate. In the recent 10-year Plan for FASD in BC [51], there is an exclusive focus on ‘women’ and ‘cultural and ethnic groups’ as populations of relevance in addressing FASD. IBPA highlights the need to move beyond such a priori foci (for which approaches such as GBA and cultural sensitivity have been criticized) towards relational understandings of such categories. Reinforcing the discourse of at-risk women or cultures perpetuates the assumption that substance use and FASD are experienced in homogenous ways within these groups. This ignores the evidence that both women and certain ‘cultural groups’ are differentially affected by substance use and FASD due to their shifting and intersecting social locations. For instance, the majority of women who have a child diagnosed with FASD also experience poverty; a fact that is often ignored in dominant FASD discourse. An IBPA unpacks ‘one-size fits all’ assumptions of policy problems and their impact on particular populations, highlighting that such assumptions risk reinforcing essentializing and discriminatory responses to particular people. This also promotes the urgent need to fill the gap in current knowledge/evidence about how substance use and FASD occurs and affects people across intersecting social locations.

Beyond bringing attention to the intersecting social locations that situate substance use and FASD, IBPA also highlights the processes of power that shape such experiences. For instance, FASD-related policy has often sought to address the social determinants or individual ‘risk factors’ situating maternal substance use, such as housing, nutrition and stress. Yet, without contextualizing such determinants as produced within proximal and systemic power dynamics (e.g., the racialization of poverty, gendered violence, etc.), the ‘problem’ becomes located within particular women, reinforcing reductive understandings and responses to ‘problem’ populations. For instance, highlighting FASD as predominantly being an issue of Aboriginal women, while failing to address the intersecting processes of power that can situate substance use (e.g., socioeconomic discrimination, neocolonialism, racialization, criminalization, etc.) serve to construct and stigmatize Aboriginal people as a problem population, reinforcing the conditions creating inequity.

### Transformative effects

The transformative thrust of IBPA can allow for policy analysis to move beyond naming inadequacies in policy towards reforming them to better reflect the differential experiences of populations and in turn, improve relations of inequity. While the *descriptive* questions employed in this case study set the stage for improving understandings and responses to maternal substance use and FASD, the *transformative* questions seek to answer the ‘how’ question. For instance, the first Transformative Question asks: *What inequities actually exist in relation to the problem?* With respect to FASD-related research and policy, this question must be asked and better addressed in order to broaden conceptions of the problem, overturn discriminatory constructions, and better address the relations of inequity that often situate understandings of and responses to substance use and FASD. Some key ‘action steps’ that can be taken in this regard include:

- promoting reflexivity and critical dialogue surrounding what is ‘known’, why, and whose interests are served with respect to current FASD research, policy and practice. This involves actively resisting moralizing and discriminatory conceptions of ‘problem holders’ which reinforce relations of inequity;
- meaningfully integrating diverse knowledges and experiences of those affected by maternal substance use across intersecting social locations within policy processes to better reflect the intersectionality of FASD.
- better accounting for the range of intersecting processes that can affect maternal substance use and FASD – research and analysis within and across shifting social locations – while placing the importance of power “front and centre” throughout such work [52].

### Case 4: Policy processes surrounding the Kelowna Accord

Despite the implementation of many health policies aiming to improve the health of Aboriginal people, inequities affecting Aboriginal people in Canada continue to increase, as illustrated by Indigenous peoples’ longstanding disproportionate burden of: infectious and chronic disease; mental health problems and suicide; substance use, trauma and violence; and inequitable access to housing, education, employment, food security and health care [53]. These health inequities are deeply tied to the history of colonialism in Canada and addressing such health inequities at their root thus calls for new ways of analyzing Aboriginal health policy issues that attend to underlying structural inequities [54]. With its attention to structural relations of power, intersectionality provides a useful theoretical lens for analyzing Aboriginal health policy issues with a view to addressing inequities.

### Structural innovations

The flexibility of IBPA allows the analyst to tailor the analysis to fit the policy problem being examined. For example, in this policy case study, the analysis relied primarily on the guiding principles and the most relevant IBPA questions; the flexibility of the Framework meant that not every question had to be answered. This was especially important for tailoring the Framework to support an analysis of policy processes, instead of the content of a particular policy. As an example of this tailoring, *descriptive* question 4, *How are groups differentially affected by this representation of the problem?* was reframed to read *How are groups differentially affected by their representation in the policy process?* Tailoring the Framework to suit analysis of policy processes, as opposed to content, illustrates how IBPA can serve as a framework for analyses that expand the boundaries of what is typically analyzed in policy analysis. Broadening the spectrum of what can be analyzed enables an analysis of various aspects of policy that are often taken for granted, such as the policymaking process. Consequently, this expanded approach to policy analysis has the potential for arriving at recommendations that are relevant beyond the scope of a single policy issue; rather the insights gained from IBPA may inform various aspects of policy and policymaking.

IBPA also provides structured guidance for applying critical perspectives to policy analysis. For example, the question, *What knowledge, values and experiences do you bring to this area of policy analysis?* prompts analysts to be transparent about their own held assumptions and political motivations, which are important given the overt political orientation of much critical policy analysis [55]. By providing a structure for articulating the political orientation of policy analysis, which is essential for ensuring rigor and scientific integrity [56], the structure of IBPA helps to ensure the rigor of critical policy analysis as well transparency in how policy solutions are reached. IBPA thus makes a significant contribution to the critical policy literature, which contains many applications of critical policy analysis, yet few that provide a detailed articulation of how critical policy analysis is done and how rigor in this form of analysis is achieved.

Unlike conventional “context-stripping” approaches to policy analysis where policy problems are typically analyzed in isolation of broader social and political contexts [57], the IBPA Framework provides a deepened contextual analysis, which can be useful for identifying underlying assumptions in the way policy problems are defined, including the way policy problems historically, politically and socially construct groups of people. For example, in this case study IBPA is used to unpack assumptions within the Kelowna Accord’s focus on the “gap between Aboriginal and non-Aboriginal Canadians” [58]. The IBPA principle of *Intersecting Categories* challenges the assumption that Aboriginal and non-Aboriginal Canadians are two neatly defined and mutually exclusive groups positioned at opposite ends of the health and social spectrum. The IBPA-informed questions prompt the analyst to think about how the policy problem might be reframed in a way that challenges such assumptions and considers social and historical contexts. IBPA, for example, might lead to a reframing of the policy problem in the Kelowna Accord as “addressing structural barriers to Indigenous peoples’ health”, which draws attention to the root causes of health inequities rather than differences between Aboriginal and non-Aboriginal people. Additionally, the IBPA tailored question, *How are diverse groups differentially affected by their representation in the policy process?* and the IBPA question, *How have representations of the problem come about?* prompt the analyst to consider how a history of intersecting oppressive systems such as colonialism, sexism and racism, operate through policies to produce layers of inequity across a spectrum of people with diverse identities.

Another example of how IBPA provides a deepened contextual analysis is by providing questions to help unpack the assumptions behind key concepts used in policymaking. In this policy case study, IBPA is used to unpack assumptions within the notion of collaboration. An IBPA approach draws attention to the social and historical context of Aboriginal health policymaking in Canada and enables a critical examination of how collaboration has occurred in policymaking. An IBPA-informed question might be, *How has collaboration been historically constructed within policy processes and what assumptions underlie these constructions?* IBPA reveals that although collaboration between governments and Indigenous leaders was a key component of the agreements reached in the Kelowna Accord, the ultimate federal government decision to not fund the proposed policies is reflective of inherent power inequities within such “collaborative” policymaking processes. In challenging key policy concepts such as collaboration within policy processes, IBPA can generate understandings that provide insight into improving policy processes, such as insights into what constitutes effective collaborative policymaking.

### Transformative effects

The IBPA *transformative* questions help to structure an analysis that arrives at action-oriented policy recommendations to address structural inequities. While other forms of critical policy analysis often result in a detailed description of the complexity of power inequities, IBPA facilitates the analyst in arriving at actionable policy recommendations that aid in transforming social structures. For example, this policy case study drew on the IBPA principle of *Diverse Knowledges* in order to focus on how diverse Indigenous peoples and knowledges were included in the Kelowna Accord policymaking processes, and how policymaking processes could be transformed to foster meaningful inclusion in the future. Including Indigenous people and knowledges in policymaking is an important step towards transforming and decolonizing policymaking processes [59].

Action-oriented policy responses are an essential part of decolonizing work, thus the transformative nature of IBPA makes it a useful decolonizing approach or methodology for policy analysis. However, the IBPA *description* questions also contribute towards the Framework’s decolonizing potential. For example, the *descriptive* questions may help to identify colonial assumptions within the definition of the policy problem and to reframe the policy problem in ways that not only resist such assumptions but also are further grounded in Indigenous perspectives. Including Indigenous peoples and perspectives in the definition of policy problems is an essential step towards self-determination and decolonization [60], and is also necessary for developing policies that address health inequities at their core.

### Case 5: Building transformative anti-colonial policy processes: lessons from an Indigenous Intersectionality-Based Policy Analysis

Provincial, national and international trends demonstrate increasing criminalization and medicalization of Indigenous girls. Indigenous youth are overrepresented in the child protection system and within the justice system of Canada [61]. In this case study, an IBPA was applied to examine historical and current construction of Indigenous girls and structural violence done through policy, and specifically the British Columbia Child and Youth Mental Health Plan [62]. The plan was the first of its kind in Canada, specifically focused on addressing underserved populations, in particular Indigenous children and youth.

The case study is written from the author’s reflexive position as a woman of Metis ancestry and part of the Secwepemc community, as a social worker, trauma therapist and activist who has directly witnessed the ineffectiveness of policies such as the British Columbia Child and Youth Mental health Plan (CYMH) in addressing the intersecting vulnerabilities of Indigenous girls. The author argues that, “I have also seen how the policy itself has in fact constructed this vulnerability, which I maintain is a form of state structural violence. Such violence occurs in the failure to act and/or in interventions of the state, via policies and systems, that lead to a culturally unsafe environment for Indigenous girls and to further violence” [63]. The case study reveals how policies not only fail to protect Aboriginal girls from victimization, but actually contribute to this victimization in many cases. It underscores that in order to understand the violence today experienced by Aboriginal girls and women, it is necessary to situate this violence within the violence of colonization, and particularly within the intersection of policies such as the *Indian Act* and other federal and provincial policies such as child welfare and youth justice policies.

### Structural innovation

The most useful aspect of the IBPA Framework for examining the Child and Youth Mental Health Plan is the set of *descriptive* questions about representations of the ‘policy problem’, in this case, violence against Indigenous girls. These questions investigate how a problem is framed, by whom and why (questions 2 and 3); what groups are most affected (question 4); and current policy responses that maintain inequities (question 5). These sets of questions provide an important starting place for policy development because they advance new understandings of violence against Indigenous girls and the mental health and wellbeing of Indigenous girls by focusing attention to the often overlooked intersections of age, geography, gender-expression and Indigeneity. An IBPA analysis also locates the source of the girls’ challenges within structural and systemic problems such as colonialism and neo-colonialism, including racism, poverty, sexism and the intersections of these in her life.

However, the greatest challenge for intersectionality, and indeed for IBPA policy analysis, is the relationship to colonization of Indigenous peoples worldwide. Given the historic and ongoing colonization of Indigenous nations within Canada, and other countries such as Australia, New Zealand, and the United States, together with post-colonial and transnational issues of colonization impacting policy throughout the world, colonialism needs to be critiqued as a central component of any policy while at the same time, resisting any kind of essentialization of Indigenous experience. So while IBPA is important for attending to many intersecting factors, including gender, sexuality, geography, age, and because it advances a commitment to social change, it does not centre Indigenous sovereignty. Until intersectionality acknowledges its own colonial history it is not well situated to address the challenges that Indigenous communities experience, in particular, violence against Indigenous girls.

This case study therefore calls for an Indigenous IBPA that is intersectional, inherently activist, responsive to local and global colonization forces, and theorized for the emergent “multifarious, polyvocal” Indigenous identity within a clear goal of sovereignty [64]. To do this, the author develops an Indigenous IBPA (IIBPA) situating mental health and trauma among Indigenous girls who have experienced violence within a broader context and acknowledging their resistance and agency at the intersection of colonialism, poverty, patriarchy, racism and discrimination, among other systems. This expanded approach understands and locates Indigenous policy analysis within the context of colonialism, past and current, and within community and relationships within the community.

### Transformative effects

Centering colonization, sovereignty, agency and resistance through an expanded Indigenous IBPA framework, leads to the recognition of the multi-generational impact of colonization and trauma and points towards policy solutions that acknowledge sovereignty, build on resistance and emerge from the strengths within the community and within girls themselves. Indigenous girls and women are the best guides of determining their own needs in this respect, as they are already engaging in daily acts of understanding, negotiating and resisting colonial policy. Numerous examples of such capacity and strength are highlighted by examples including survivance stories of Angel Streets, the film *Highway of Hope* [65]*,* Indigenous girls groups and in individual Indigenous girls’ stories.

An IBPA within an Indigenous framework understands the diversity that exists within communities and across Indigenous cultures. An Indigenous IBPA (IIBPA) argues for policy processes to be rooted in a deep awareness of the forces of colonial oppression, past and present, situated and developed in the local Indigenous community and knowledge, and include a holistic understanding of health policy as including mental, spiritual, physical and emotional, and would build on the strengths and resistance that exist within Indigenous communities, blending traditional and contemporary approaches. And, by focusing on the agency of individual Indigenous girls and women, the implementation of an IIBPA would support the development of more ethical, anti-colonial and ultimately less violent policies for dealing with violence against Indigenous girls.

### Case 6: HIV testing and the criminalization of HIV non-disclosure

To the dismay of many public health actors, the Supreme Court of Canada recently ruled that that the duty for an individual with HIV to disclose her/his serostatus can be dispensed only when: a condom is used and the individual has a low viral load [66]. This case study helps to illuminate some of the reasons that the recent decision is regressive and highly dangerous from a public health and equity perspective. It examines the possible relationship between innovations in laboratory technologies that can detect HIV during early stages of infection and the increasing use of the criminal law to prosecute alleged cases of HIV non-disclosure in Canada. The case study argues that both targeted HIV testing initiatives and the prosecution of alleged HIV non-disclosure cases in Canada ignore the structural drivers of the epidemic and problematically conceive of the ‘problem’ which must be addressed. The analysis has international implications given the growing trend globally to criminalize people living with HIV in cases of HIV non-disclosure where exposure and/or transmission occurs [67].

### Structural innovation

The flexible nature of the IBPA Framework allowed a multilevel analysis to be conducted across two complex policy domains. An IBPA reveals not only the unintended effects that policies may have on differentially situated actors (for example, the ways in which HIV-disclosure may be particularly difficult for some groups of women) but also the unintended effects health and health-related policy responses may have upon one another for example, how a culture of criminalization may serve as a deterrent to getting tested for HIV). By considering complex public health issues together, key tensions can be identified *within* and *across* different health and health-related policy areas. This exploratory IBPA provides both in-depth, historically situated analysis of these policy domains as well as summary tables that concisely review key issues both separately and in relation.

Critical analysis reveals the importance of reflecting on the idea of *standpoint* when thinking about the ‘value added’ of the IBPA in three interrelated respects. First, an IBPA accounts for the standpoint of the policy actor/researcher performing the analysis. Conducting an IBPA demands ‘doing’ reflexivity and accounting for one’s intersectional standpoint and the place from which one views a policy issue. Second, the notion of standpoint is important in considering the range of actors (or standpoints) that should be engaged when conducting an IBPA and the diverse sources of evidence need to get a robust picture of the policy problem. Third, the conception of standpoint helps to elucidate the imagined standpoints and subject positions of persons within policy. As reviewed, policies have the ability to ‘create’ people and an IBPA helps to reveal the possible disjunctures between imagined/constructed standpoints within policies and the everyday actualities of persons who sit at varied axes of oppression and marginalization.

### Transformative effects

IBPA underscores how building on the lived experiences and knowledges of persons has transformative potential and is central to thinking about how policy actors use categories of ‘most-at-risk populations’ (MARPs) in policy strategies—e.g., what groups like ‘gay’, ‘MSM’ (men who have sex with men) or ‘Black MSM’ may reveal and/or erase. Building on this point, the author argues:

Intersectionality can help make visible the kinds of mutually constituting intersections that must be considered in complex policy fields…an IBPA demands that policy actors consider the complex, dialectical nature between systems of penalty and privilege and the individuals and groups who have intersectional standpoints along various social identities and lived actualities [68].

The generation of these new, equity-focused perspectives is a key advantage to intersectional thinking.

While testing is an important albeit insufficient aspect of HIV-prevention efforts, this analysis demonstrates the ways in which the increasing trend towards criminalizing HIV non-disclosure cases in Canada poses significant public health challenges for mobilizing an effective response to the epidemic. As noted above, using an IBPA allowed for an exploration of why HIV/AIDS policies and governmental strategies must be understood as relational processes. Further, this IBPA provides an explication of the ways in which medical technologies have significant implications for sexuality and the law across diverse policy fields.

Echoing the analysis advanced in this case study, civil society groups internally have been working to underscore the many reasons why the “creep of criminalization” is problematic and highly stigmatizing for people living with HIV [66]. This IBPA engages with current advocacy efforts in Canada and internationally to illuminate the advocacy strategies used and challenges faced by actors seeking to challenge and transform dominate modes of disease governance. Placing analytic attention to these efforts, such as the campaign for prosecutorial guidelines in Ontario, Canada reviewed by Grace, creates an opportunity to consider opportunities for coalition building and intersectoral action.

### Case 7: Funding of gay men’s HIV prevention

For three decades, gay men have remained a key population dramatically impacted by HIV in the province of British Columbia. However, despite this well documented inequity, policies and investments to support prevention activities among this population have generally fallen short. An audit conducted in 2001 concluded that only 1% of the HIV funding went for gay men’s prevention [69]. This neglect was subsequently reported by activists, researchers and policy makers [70] – however, there has been little discussion to why this state of neglect is allowed to persist as gay men continue to account for over half of the HIV infections in the region [71]. This case study applied the IPBA Framework to explore the current state of funding and identified the processes and key issues that prevent adequate funding for HIV prevention with gay men.

### Structural innovation

This review, like previous ones, demonstrated a lack of investment in gay men’s HIV prevention, however the IBPA Framework was useful in identifying some issues that were not raised in previous analysis and discourses on HIV prevention funding. These issues were revealed through qualitative interviews with key informants that were guided by the Framework’s questions. The IBPA questions were also carefully adapted to the specificity of the topic to guide the analysis.

Working through the questions from the Framework helped identify some key tensions in the funding allocation process for HIV prevention. One of these tensions was identified by the Framework’s attention to diverse knowledges. Indeed, there were dramatic differences between the community and public health’s definition and understanding of HIV prevention. While, the public health definition emphasizes clinically based approaches such as the expansion of testing and treatment, community described prevention as the promotion of health and wellness from a holistic and right-based perspective. When reviewing funded initiatives, the vast majority of the interventions subscribed to the public health definition of prevention; with most prevention dollars for gay men going to activities related to HIV testing. However, research and observations to date suggests that a singular focus on testing and treatment is unlikely to resolve the epidemic among gay men [72]. Other strategies must be promoted to reduce gay men’s inequities in terms of HIV infection, including community led initiatives since they have been generally much more successful at reducing HIV transmission than public health interventions [73].

The IBPA Framework also helped reveal multiple assumptions behind the distribution of HIV prevention funding. For example, it is often assumed in prevention that gay men form a monolith and that the “at risk populations” described by public health (such as gay men, injection drug users, Aboriginal population etc.) are all-distinct. The IBPA Framework emphasizes that individuals and communities are constituted of multiple and interacting social locations and that therefore may belong to multiple “at risk” categories and therefore can potentially find themselves at greater risk of HIV infection. However, when reviewing currently funded initiatives, none address gay men who belong to multiple “subordinate” or “at risk” group such as gay men of colour, Aboriginal gay men, gay men in prison, gay men who inject drugs. These groups tend to be left without any interventions, therefore increasing inequities within the gay community.

### Transformative effects

The common explanation from gay men’s advocate has generally been that homophobia, and homophobia alone, is the cause of the lack of resources for HIV prevention. However, the application of the IBPA revealed a pattern of systemic discrimination against gay men that is defined at the intersection of heterosexism, medicalization of prevention and sex panic. The application of the Framework’s questions showed a complete lack of funded interventions that address the sexual health needs and sexual rights of gay men – in fact, there was evidence that governments refrain from funding sexualized interventions. The increased support for medical intervention as noted by this analysis may be directly linked to the discomfort of governments and public health institutions at being perceived as supporting homosexuality or sexualities they see as perverted.

By illuminating these factors and providing a new perspective on the factors preventing funding for HIV prevention, the IBPA Framework can help propose radically different solutions for advocacy and to reverse the situation. Several scholars have noted that intersectionality has the potential to help identify less obvious similarities among populations and groups that can lead to coalition building [74,75]. In this case, gay men have generally been alone within the HIV movement to denounce homophobia within governments. However, this isolation could shift if the focus is diverted away from the subordination associated with a gay identity and towards a focus on sexual rights that intersect with gay health, but also Indigenous health, women’s health, etc. Gay men may have been mostly alone to cope with the impacts of homophobia within the AIDS infrastructures, but other groups have suffered of moralistic views on sexuality with whom gay advocates could partner to see their sexual rights promoted within the HIV field.

## Conclusion

In this paper we aim to expand current paradigms of policy analysis by introducing an IBPA Framework and importantly, demonstrating its worth in a variety of health related policy areas. The case studies strive to bring issues of equity to the fore and ultimately inspire other policy practitioners and researchers to use this approach in their own policy work. While the examples here show the potential and significance of operationalizing intersectionality, it is important to note that the IBPA Framework is not without its challenges.

First, the very process of implementing such an approach can be resisted by those who are not open to social justice oriented change and/or asking difficult questions about power and structural asymmetries in the context of politics and policy. Second, even among those committed to such change, the IBPA may be rejected for its purposeful movement away from prioritizing - a priori - certain factors, often seen as central to shaping inequities, such as gender or Indigenous sovereignty and resistance, and instead leaving the determination of what is important to the process of discovery. Third, new types of expertise are required to move beyond the status quo of specifically focusing on single or even additive approaches (e.g., gender + age + race) and instead capturing multiple and intersecting locations and social structures. Often the evidence required for an IBPA application is either absent or in very nascent stages of existence. Related to this is the challenge of ensuring that when possible all relevant lived positions in relation to a policy problem or priority are captured and that in the process, appropriate types of data are collected and analyzed.

As illustrated by the diverse case studies in this paper, researchers chose which IBPA questions to focus on. While providing important flexibility, this flexibility also raises the issue of whether something was missed from the final analysis because of the avenues of inquiry that were chosen or alternatively left out. And finally, even if the IBPA is rigorously applied and new ways of thinking about a policy problem or issue are revealed there still remain obstacles in terms of translating complex knowledge into accessible condensed messages for policy actors to digest and understand. Ultimately there are no guarantees that such critical research will lead to action or more precisely structural change. Processes of social transformation have to involve many kinds of interventions, actions and actors, including but not limited to the realm of policy analysis.

Nevertheless, the IBPA Framework, as demonstrated by the case studies presented here, is an innovative mechanism for analyzing the operation of power and processes of stigmatization in policy making. It is important to highlight that the architects of the IBPA envisioned it to be a living document that will change and evolve over time as a range of end users pilot test and provide feedback on how the Framework can be improved and made more practical, effective and precise. The IBPA Framework and case studies presented in this paper are thus a first step in contributing to the emerging literature in the field, expanding current paradigms of policy analysis, and allowing policy actors to see themselves as critical and potentially transformative players in the development, implementation and evaluation of policy.

### Endnotes

^a^For a detailed discussion and comparison of key current equity-focused policy analysis tools used to capture the differential effects of policy on the population in Canada, including sex and gender based analysis (SGBA) and health and health equity impact assessments (HIAs/HEIAs), see Hankivsky et al. [22].

^b^The IBPA Framework contains sub-questions relating to each overarching question to help guide analyses. Please see Hankivsky [11] for more details.
